# IL-13 may be involved in the development of CAD via different mechanisms under different conditions in a Chinese Han population

**DOI:** 10.1038/s41598-018-24592-9

**Published:** 2018-04-18

**Authors:** Ling-Feng Zha, Shao-Fang Nie, Qian-Wen Chen, Yu-Hua Liao, Hong-Song Zhang, Jiang-Tao Dong, Tian Xie, Fan Wang, Ting-Ting Tang, Ni Xia, Cheng-Qi Xu, Ying-Chao Zhou, Zhi-Peng Zeng, Jiao Jiao, Peng-Yun Wang, Qing K. Wang, Xin Tu, Xiang Cheng

**Affiliations:** 1 Department of cardiology, Union Hospital, Tongji Medical College, Huazhong University of Science and Technology, Wuhan, 430022 China; 2 Key Lab for biological targeted therapy of Education Ministry and Hubei province, Union Hospital, Tongji Medical College, Huazhong University of Science and Technology, Wuhan, 430022 China; 3 Department of Biostatistics and Epidemiology, University of Pennsylvania, Philadelphia, PA 19104 USA; 4 Key Laboratory of Molecular Biophysics of Ministry of Education, College of Life Science and Technology, Center for Human Genome Research, Cardio-X Institute, Huazhong University of Science and Technology, Wuhan, 430074 China

## Abstract

Interleukin-13 (IL-13) has important functions in atherosclerosis, but its role in coronary artery disease (CAD) is unclear. Here, we studied the genetic role of IL-13 in CAD in a Chinese Han population using tag SNPs covering the whole *IL13* gene (i.e., rs1881457, rs2069744 and rs20541) and a two-stage cohort containing 1863 CAD cases and 1841 controls. Traditional risk factors for CAD, such as age, BMI, and other factors, were used as covariates in logistic regression analysis. In the total population, we found that two haplotypes of *IL13* (ATG and ATA, ordered rs1881457^C^-rs2069744^T^-rs20541^A^) significantly contributed to the risk of CAD with adjusted *p* values less than 0.05 (*p*_adj_ = 0.019 and *p*_adj_ = 0.042, respectively). In subgroup population analyses, the variant rs1881457^C^ was found to significantly contribute to a nearly two fold increase in the risk of CAD in men (*p*_adj_ = 0.023, OR = 1.91, 95% CI: 1.09-3.33). The variant rs1881457^C^ also significantly contributed to a nearly twofold risk of late-onset CAD (*p*_adj_ = 0.024, OR = 1.93, 95% CI: 1.09-3.42). In conclusion, *IL13* might be involved in CAD via different mechanisms under different conditions in the Chinese Han population.

## Introduction

Previous meta-analyses and genome-wide association studies (GWAS) have detected approximately 95 risk variants for CAD, but the substantial heritability of CAD remains unclear^1–3^.

Interleukin-13 (IL-13) was co-discovered by Minty *et al*. and McKenzie *et al*. as a new inflammatory cytokine in 1993. IL-13 is expressed in activated human T lymphocytes and has been reported to play important roles in inflammatory and immune responses^4,5^. In 2003, Gordon demonstrated that IL-13 could alternatively activate macrophages, which have different roles in humoral immunity and repair^6^. Early studies reported that IL-13 can increase CD36 expression through PPARγ activation and increase macrophage foam cell formation, which might then lead the development of atherosclerosis^7–9^. However, later studies demonstrated that IL-13 can maintain metabolic homeostasis by inducing macrophage PPARδ or PPARβ expression in adipose tissue, which might then promote the development of atherosclerosis^10,11^. Additionally, in 2012, Cardilo-Reis *et al*. performed *in vivo* studies and found that IL-13 inhibits the progression of atherosclerosis through a new mechanism that is not related to cholesterol levels^12^. In 2013, Stanya *et al*. demonstrated that IL-13 deficiency in mice leads to increased weight gain, hyperglycemia, and hepatic insulin resistance, which indicates that IL-13 might inhibit atherogenesis^13^. Jiang *et al*. found that IL-13 is involved in glucose uptake and metabolism in skeletal muscle, which regulates glucose homeostasis in metabolic diseases^14^. These findings indicate that IL-13 might be involved in atherosclerosis and has complex functions. Most recently, Amit *et al*. studied the function of IL-13Rα1 in myocardial homeostasis and heart failure and discovered that IL-13 is up-regulated in diabetic hearts^15^. Concordantly, Stanya *et al*. reported that IL-13 can control hepatic glucose production^13^, which might cause the reactivity in up-regulation of IL-13 in the diabetic heart.

In previous GWASs, such as analyses from the CARDIoGRAMplusC4D Consortium, two SNPs localized in the *IL13* region were found to have no association with CAD and/or myocardial infarction (*p* = 0.260 for rs20541 and *p* = 0.975 for rs2069744). However, these data sets were mainly based on a European population, and the reported SNPs did not cover the whole *IL13* region (UCSC). Additionally, a promoter variant, i.e., rs1881457, of *IL13*, which has not been studied in previous CAD GWASs, has been reported to regulate *IL13* expression and might be an important functional variant in terms of the risks of various diseases^16,17^.

Therefore, here, we first selected tag variants covering the whole *IL13* gene that included both common coding and regulatory variants and then tested the genetic associations between the selected tag variants of *IL13* and CAD in a two-stage case-control cohort from a large Chinese Han population. Next, we classified the total studied population into different subgroups and tested the associations between the selected variants and CAD under different conditions. Subsequently, we assessed CAD severity using the Gensini score and examined whether the selected variants influenced this parameter. These data may elucidate the genetic function of IL-13 in CAD, which may help to determine the exact role of IL-13 in CAD.

## Results

### Populations

In stage 1, i.e., the discovery cohort, the CAD cases were found to have higher BMI, TG, LDL-c, and Tch level and to be older than the control subjects (Table 1). Smoking, male gender, diabetes mellitus and hypertension were more prevalent among the CAD cases than the controls. The CAD patients had lower levels of HDL-c compared with the control individuals. The validation and combined populations had the same population characteristics. Our sample size provided more than 80% statistical power for the genetic association studies conducted here.

**Table 1:** The populations’ characteristics.

Characteristics	Discovery cohort		*p*	Validation cohort		*p*	Combined cohort		*p*
	CAD (n = 768)	Control (n = 768)		CAD (n = 1095)	Control (n = 1073)		CAD (n = 1863)	Control (n = 1841)	
Age (years)	63.33 ± 11.05	51.61 ± 12.33	<10^−6^	61.73 ± 11.35	51.55 ± 12.79	<10^−6^	62.39 ± 11.25	51.58 ± 12.59	<10^−6^
Male (%)	71.48	59.24	<10^−6^	73.61	65.52	4.2 × 10^−5^	72.73	62.90	<10^−6^
Smoking (%)	46.48	20.18	<10^−6^	44.47	5.87	<10^−6^	45.30	11.84	<10^−6^
BMI (kg/m^2^)	24.24 ± 1.54	23.66 ± 1.41	<10^−6^	24.37 ± 1.60	23.74 ± 1.42	<10^−6^	24.32 ± 1.58	23.71 ± 1.41	<10^−6^
Hypertension (%)	68.62	14.71	<10^−6^	67.21	1.12	<10^−6^	67.79	6.79	<10^−6^
DM (%)	34.77	4.30	<10^−6^	30.59	0.09	<10^−6^	32.31	1.85	<10^−6^
Tch (mmol/L)	5.09 ± 1.18	4.53 ± 0.90	<10^−6^	5.15 ± 1.18	4.88 ± 0.84	<10^−6^	5.13 ± 1.18	4.73 ± 0.88	<10^−6^
TG (mmol/L)	1.79 ± 1.16	1.45 ± 0.87	<10^−6^	1.83 ± 1.31	1.53 ± 1.06	<10^−6^	1.81 ± 1.25	1.49 ± 0.99	<10^−6^
HDL^−^c (mmol/L)	1.12 ± 0.29	1.28 ± 0.31	<10^−6^	1.11 ± 0.28	1.42 ± 0.26	<10^−6^	1.11 ± 0.29	1.36 ± 0.29	<10^−6^
LDL-c (mmol/L)	2.98 ± 1.02	2.54 ± 0.72	<10^−6^	3.06 ± 1.09	2.76 ± 0.77	<10^−6^	3.03 ± 1.06	2.67 ± 0.76	<10^−6^

The data are provided as the mean ± SD. Categorical data, including gender, smoking status and other data, were tested using chi-square tests, and measurement data, such as BMI, age and blood lipid levels, were tested using *t*-tests between the cases and controls in each population; Age for the case group is the age at diagnosis; age for the control group is the age at enrollment. CAD, coronary artery disease; BMI, DM, diabetes mellitus; body mass index; Tch, total cholesterol; HDL-c, high-density lipoprotein cholesterol; TG, triglyceride; LDL-c, low-density lipoprotein cholesterol.

### Association analysis of the variants in *IL13* with CAD in a Chinese Han population

The *p* values from Hardy-Weinberg disequilibrium tests in the control subjects were more than 0.001 for all of the studied variants. In the allelic association analysis, we found the following: in the discovery cohort, none of the three variants (i.e., rs1881457, rs2069744 and rs20541) in *IL13* exhibited a significant association with CAD before or after adjustment for the traditional risk factors for CAD (*p* > 0.05); in the validation cohort, the three variants also exhibited no associations with CAD (*p* > 0.05); and in the combined cohort, the association results between all three SNPs and CAD were non-significant (*p*_adj_ > 0.05; Table 2).

**Table 2:** Allelic association analysis of the variants in *IL13* with CAD in the Chinese Han population.

Population	SNP allele	MAF		*p*_hwb_	*p*_obs_	*p*_adj_	OR (95% CI)
		Case	Control				
Discovery cohort	rs1881457^C^	0.267	0.245	0.283	0.175	0.475	1.10 (0.85–1.40)
	rs2069744^T^	0.096	0.106	0.345	0.422	0.433	0.86 (0.59–1.25)
	rs20541^A^	0.309	0.332	0.298	0.224	0.286	0.87 (0.67–1.13)
Validation cohort	rs1881457^C^	0.239	0.246	0.507	0.640	0.421	0.87 (0.62–1.22)
	rs2069744^T^	0.076	0.093	0.509	0.088	0.582	0.86 (0.50–1.47)
	rs20541^A^	0.312	0.334	0.009	0.167	0.671	0.93 (0.67–1.29)
Combined cohort	rs1881457^C^	0.250	0.245	0.223	0.630	0.370	1.09 (0.90–1.31)
	rs2069744^T^	0.085	0.098	0.832	0.076	0.381	0.88 (0.66–1.18)
	rs20541^A^	0.311	0.333	0.006	0.061	0.361	0.92 (0.76–1.11)

CAD, coronary artery disease; SNP, single nucleotide polymorphism; MAF, minor allele frequency; *p*_obs_, observed *p*-value; *p*_hwe_, *p*-value of the Hardy–Weinberg equilibrium tests; *p*_adj_, *p*-value after adjusting for age, BMI, gender, hypertension, smoking history, diabetes mellitus, LDL-c, TG, Tch and HDL-c; OR, odds ratio after the adjustment.

Based on the genotypic association analysis, rs2069744 was significantly associated with CAD in the recessive model in the discovery cohort (*p*_adj_ = 0.044, OR = 0.18, 95% CI: 0.04-0.96). However, in the validation and combined cohorts, the association with rs2069744 did not remain significant (*p*_adj_ > 0.05; see Supplementary Table S1). The other two variants also exhibited the same non-significant association with CAD in all of the studied cohorts (*p*_adj_ > 0.05; see Supplementary Table S1).

Additionally, we analyzed the haplotypic association between *IL13* and CAD in the combined population. Two haplotypes (ATG and ATA, ordered rs1881457^C^-rs2069744^T^-rs20541^A^ in *IL13*) were significantly associated with CAD (*p*_adj_ = 0.019 for ATG and *p*_adj_ = 0.042 for ATA; see Supplementary Table S2). We further performed an association analysis of the haplotypes and Gensini scores in the CAD patients but found no significant association result (*p*_adj_ = 0.818). Unfortunately, the numbers of these haplotypes were too small to reach a statistical power of 80%.

### Association analysis of the selected variants in *IL13* with CAD in the subgroup populations

First, we separated the CAD patients into two subgroups, i.e., an anatomical-CAD group (CAD patients with anatomical disease i.e., severe coronary stenosis) and a clinical-CAD group (patients with clinical disease, i.e., MI or revascularization). In both the anatomical-CAD and clinical-CAD subgroups, the allelic association analyses revealed no significant associations of any of the SNPs with CAD after adjustment for the traditional risk factors (*p*_adj_ > 0.05; see Supplementary Table S3). Genotypic association analysis also demonstrated that the three SNPs were not associated with CAD in either subgroup (see Supplementary Table S3).

Second, we divided our sample into two subgroups by gender to investigate the associations of the three SNPs (rs1881457, rs2069744 and rs20541) with CAD in the different genders. In the female subgroup, none of the three SNPs exhibited a significant association with CAD in the allelic or genotypic association analyses (Table 3). In the male subgroup, the allelic association analysis revealed no significant associations of any of the SNPs with CAD before or after adjustment for the traditional risk factors (*p* > 0.05; Table 3). However, the genotypic association analysis found a significant association between rs1881457^C^ and CAD in the recessive model (*p*_adj_ = 0.023, OR = 1.91, 95% CI: 1.09-3.33; Table 3).

**Table 3:** Association analysis between the SNPs in *IL13* and CAD in the gender subgroup populations.

Population	SNP allele	Model	Male			Female		
			*p*_obs_	*p*_adj_	OR (95% CI)	*p*_obs_	*p*_adj_	OR (95% CI)
Combined cohort	rs1881457^C^	ALLE	0.895	0.326	1.12 (0.89–1.41)	0.185	0.776	1.05 (0.76–1.45)
		ADD	0.456	0.334	1.12 (0.89–1.40)	0.445	0.788	1.04 (0.77–1.41)
		DOM	0.554	0.944	1.01 (0.76–1.34)	0.250	0.610	1.11 (0.74–1.68)
		REC	0.385	0.023	1.91 (1.09–3.33)	0.364	0.806	0.92 (0.46–1.82)
	rs2069744^T^	ALLE	0.296	0.698	0.93 (0.65–1.33)	0.148	0.303	0.76 (0.45–1.28)
		ADD	0.550	0.697	0.93 (0.65–1.33)	0.210	0.305	0.76 (0.45–1.28)
		DOM	0.276	0.822	0.96 (0.65–1.40)	0.216	0.366	0.77 (0.44–1.35)
		REC	0.892	0.419	0.53 (0.12–2.45)	0.145	0.425	0.38 (0.03–4.16)
	rs20541^A^	ALLE	0.360	0.352	0.90 (0.71–1.13)	0.028	0.780	0.95 (0.68–1.34)
		ADD	0.325	0.340	0.89 (0.70–1.13)	0.065	0.766	0.95 (0.66–1.36)
		DOM	0.177	0.594	0.92 (0.68–1.25)	0.022	0.884	0.97 (0.61–1.52)
		REC	0.831	0.231	0.73 (0.43–1.23)	0.267	0.671	0.83 (0.34–1.99)

SNP, single nucleotide polymorphism; *p*_obs_, observed *p*-value; *p*_adj_, *p*-value after adjusting for age, BMI, diabetes mellitus, smoking history, hypertension, and lipid levels; OR, odds ratio after the adjustment; ADD, additive model, rs1881457_CC/AC/AA; rs2069744_TT/CT/CC; rs20541_AA/GA/GG; DOM, dominant model, rs1881457_CC + AC/AA; rs2069744_TT + CT/CC; rs20541_AA + GA/GG; REC, recessive model, rs1881457_CC/AC + AA; rs2069744_TT/CT + CC; rs20541_AA/GA + GG.

Finally, we divided the CAD populations according to the age of onset of CAD. The patients with an age of onset of CAD of less than 55 years for males and less than 65 years for females were defined as the early-onset CAD subgroup. The other patients were defined as the late-onset CAD subgroup. No significant association results were discovered between the selected variants and CAD in the early-onset CAD group (*p*_adj_ > 0.05). However, in the late-onset CAD subgroup, rs1881457^C^ resulted in a nearly twofold increase in the risk of CAD (*p*_adj_ = 0.024, OR = 1.93, 95% CI: 1.09-3.42; Table 4).

**Table 4:** Association analysis between the SNPs in *IL13* and CAD in the onset age subgroups.

Population	SNP allele	Model	CAD early-onset		CAD late-onset	
			*p*_adj_	OR (95% CI)	*p*_adj_	OR (95% CI)
Combined cohort	rs1881457^C^	ALLE	0.704	0.95 (0.75–1.22)	0.099	1.23 (0.96–1.57)
		ADD	0.712	0.96 (0.76–1.21)	0.109	1.21 (0.96–1.54)
		DOM	0.614	0.93 (0.69–1.25)	0.396	1.14 (0.84–1.54)
		REC	0.945	1.02 (0.58–1.80)	0.024	1.93 (1.09–3.42)
	rs2069744^T^	ALLE	0.348	0.84 (0.57–1.22)	0.669	0.92 (0.63–1.35)
		ADD	0.355	0.84 (0.58–1.22)	0.669	0.92 (0.63–1.35)
		DOM	0.407	0.84 (0.56–1.26)	0.915	0.98 (0.65–1.47)
		REC	0.507	0.61 (0.14–2.65)	0.162	0.33 (0.07–1.56)
	rs20541^A^	ALLE	0.508	0.92 (0.72–1.18)	0.555	0.93 (0.73–1.19)
		ADD	0.493	0.91 (0.71–1.18)	0.541	0.92 (0.72–1.19)
		DOM	0.506	0.89 (0.64–1.24)	0.782	0.96 (0.69–1.33)
		REC	0.704	0.89 (0.50–1.59)	0.376	0.77 (0.44–1.37)

The early-onset CAD group contained subjects with onset age of CAD less than less than 65 years for females and 55 years for males. MAF, minor allele frequency; *p*_adj_, *p*-value adjusted for age, BMI, gender, hypertension, smoking history, and lipid levels; OR, odds ratio after the adjustment.; ADD, additive model, rs1881457_CC/AC/AA; rs2069744_TT/CT/CC; rs20541_AA/GA/GG; DOM, dominant model, rs1881457_CC + AC/AA; rs2069744_TT + CT/CC; rs20541_AA + GA/GG; REC, recessive model, rs1881457_CC/AC + AA; rs2069744_TT/CT + CC; rs20541_AA/GA + GG.

### Effect of rs1881457 in *IL13* on the severity of CAD

We also tested the association between rs1881457 in *IL13* and CAD severity as assessed by the Gensini scoring system. The 1^st^ (lowest) and 4^th^ (highest) quartiles of the LN of the Gensini scores were selected for the case-control association analysis. According to the allelic association analysis, rs1881457 was not associated with CAD severity (quantitative trait association, *p*_adj_ = 0.594; case-control association by the 4^th^ vs 1^st^, *p*_adj_ = 0.777, OR = 1.04, 95% CI: 0.80–1.35; see Supplementary Table S4). Additionally, we performed Mann-Whitney *U* tests to assess the influence of the rs1881457 genotypes on the severity of CAD. There were no significant associations between these variants and the Gensini scores, which was consistent with the above association results (*p* > 0.05; see Supplementary Fig. S1).

## Discussion

Here, in a Chinese Han population, we explored the genetic role of IL-13 in CAD. We found the following results: two haplotypes in *IL13* (ATG and ATA, ordered rs1881457^C^-rs2069744^T^-rs20541^A^) significantly contributed the risk of CAD; the promoter variant rs1881457^C^ of *IL13* not only contributed to the risk of CAD in male patients but was also involved in the development of late-onset CAD; and, in contrast, rs1881457^C^ did not influence the severity of CAD as determined by the Gensini scores.

**Figure 1:**
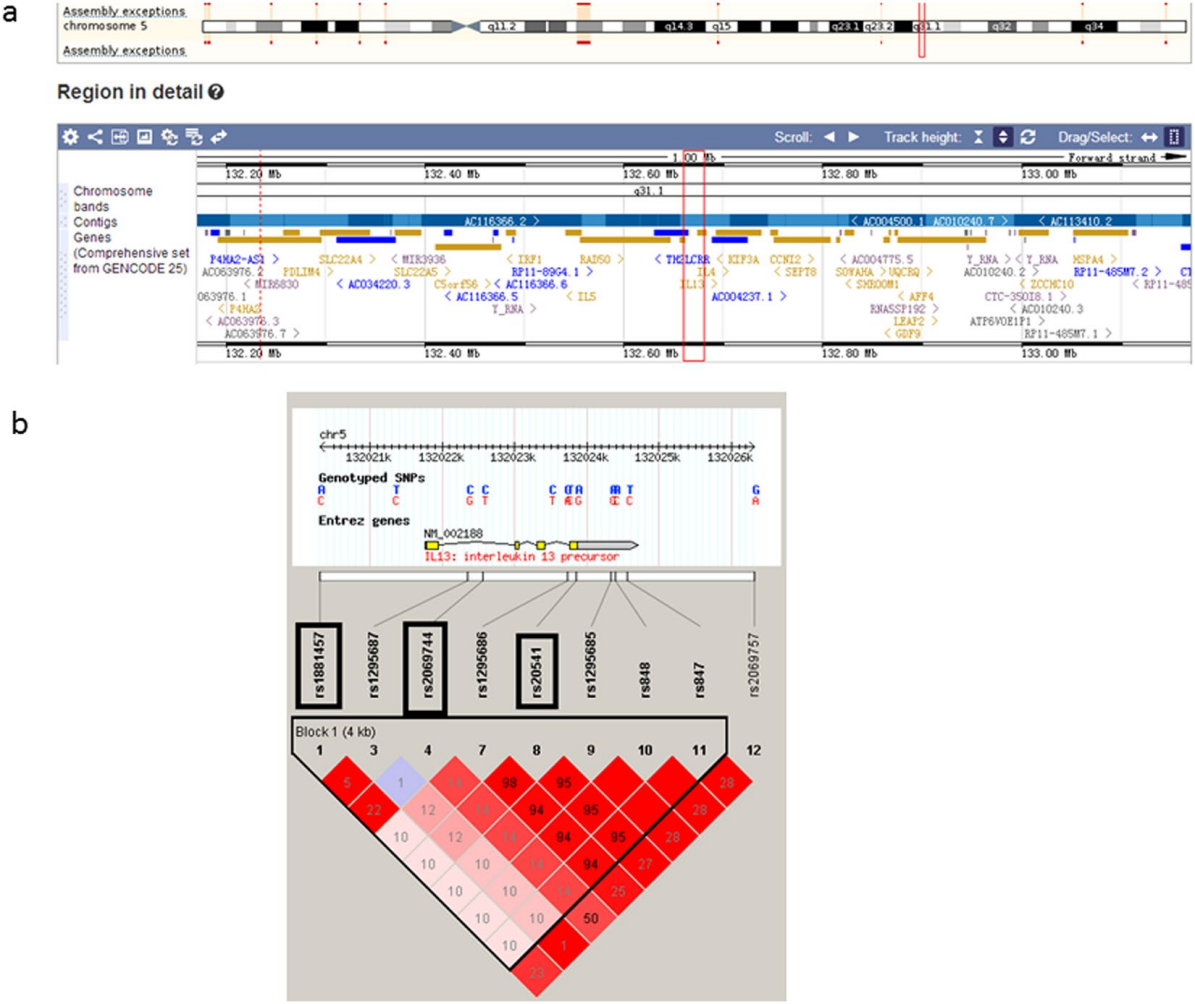
The location of *IL13* and the LD block of *IL13*. (**a**) Location of *IL13* in the Ensembl database. *IL13* is located in the chromosome 5p31.1 region with a length of 2,938 bp. (**b**) The LD block of *IL13* (HapMap CHB and JPT data sets, v.3, release 2). Each diamond indicates the LD degree between the SNPs. The number in each diamond indicates the *r*^*2*^ value. The color indicates the *D’*. Dark red regions represent a high LD. White regions represent a low LD. The black boxes show the selected tag SNPs in this study.

*IL13* is located in chromosomal region 5p31.1 near *IL4* and has a length of 2,938 bp as illustrated in Fig. 1a. Two variants, rs2706399 in *IL5* and rs273909 in *SLC*2*2A4-SLC22A5*, which have been associated with CAD susceptibility in previous studies, are located in the same region near *IL13*^18^. Previous data have confirmed that IL-13, IL-4 and IL-5 have important functions in autoimmune and inflammatory diseases^19–24^. Studies have also reported that IL-13, IL-5 and IL-4 all participate in the pathological process of atherosclerosis, which is the major pathogenesis of CAD^12,25–27^. Additionally, Newland *et al*. found that the genetic ablation of ILC2 in *Ldlr*^*-/-*^ mice accelerates atherosclerosis progression, which might be due to the loss of IL-5 and IL-13 expression by ILC2 cells^28^. Therefore, *IL13* might be involved in CAD and might be a valuable susceptibility locus for CAD.

In this study, we selected three tag SNPs in *IL13*, including rs1881457, rs2069744 and rs20541, to test the associations of these risk variants with CAD. Among these variants, rs20541 in *IL13* has been reported to be a critical genetic risk variant in asthma^29^. Therefore, we concluded that this variant might also be involved in CAD. However, we did not replicate the association between rs20541 and CAD, and we also found that one of the other SNPs, i.e., rs2069744, was not a risk factor for CAD, which is consistent with previous GWAS results from the CARDIoGRAMplusC4D Consortium. However, we found other interesting results: two haplotypes in *IL13* (ATG and ATA, ordered rs1881457^C^-rs2069744^T^-rs20541^A^) might influence the risk of CAD, and rs1881457^C^ might increase the CAD risk in the male population and might also increase the risk of late-onset CAD in the Chinese Han population. Rs1881457, which is located in the promoter region of *IL13*, was not in the same linkage disequilibrium (LD) region as rs20541 and rs2069744 and was not detected in a previous large GWAS or meta-analysis of CAD that included both European and Asian populations. Therefore, we hypothesized that rs1881457 is involved in CAD via its regulation of *IL13* expression. Recently, researchers have demonstrated that variants that regulate the expressions of various genes, which are also known as expression quantitative trait loci (e-QTL), exhibit population heterogeneity and tissue cell specificity and influence the risks of diseases in different conditions^30,31^. Rs1881457, the promoter variant of *IL13*, might be an e-QTL of *IL13*. Additionally, previous studies have reported different roles of IL-13 in vascular tissue, adipose tissue, hepatic tissue, skeletal tissue and the heart^7–11^. These findings might be due to the different e-QTLs of *IL13*, especially in different tissues under different disease conditions, which lead to the complex role of IL-13 in atherosclerosis by conditionally regulating *IL13* expression. In 2000, Patricia *et al*. found that HS4 marked the promoter region of *IL13* and then enhanced *IL13* promoter activity^32^. Moreover, these researchers also found that rs1881457 is located in the central portion of HS4 and provided a binding site for the transcription factor Oct-1, which enhances *IL13* expression^17^. Here, we discovered that rs1881457^C^ might increase the risk of late-onset CAD risk in the Chinese Han population. In 2014, Li *et al*. found that the serum IL-13 level is higher in aged healthy individuals than in young individuals^33^. These findings indicate that the minor allele “C” of rs1881457 might down-regulate *IL13* expression in the elderly population. Furthermore, Wouters *et al*. demonstrated that rs1881457 regulates the mRNA expression of *IL13* in both irritable bowel syndrome patients and controls^16^.

Cardilo-Reis *et al*. determined that IL-13 inhibits the progression of atherosclerosis in *Ldlr*^−/−^ mice^12^. Stanya *et al*. demonstrated that IL-13 deficiency in mice leads to increased weight gain, hyperglycemia, and hepatic insulin resistance^13^. Jiang *et al*. discovered that IL-13 is involved in glucose uptake and metabolism in the skeletal muscle, which regulates glucose homeostasis in metabolic diseases^14^. Most recently, Newland *et al*. demonstrated that a major innate cell source of IL-13 is required for its atheroprotective effects against the development of atherosclerosis^28^. Based on these findings, we concluded that, under specific conditions, the “C” allele of rs1881457 might increase the risk of CAD by reducing *IL13* expression. In our study, we found that the prevalence of diabetes mellitus in the late-onset CAD subgroup population was significantly higher than that in the early-onset CAD subgroup population (see Supplementary Table S5). We hypothesized that, in diabetes mellitus, rs1881457^C^ could reduce the IL-13 level and consequently increase the risk of CAD. Decreased IL-13 increased hyperglycemia and hepatic insulin resistance, decreased glucose uptake and then accelerated atherosclerosis, and this might be a slow and cumulative process. Cardilo-Reis *et al*. found that IL-13 could not reverse the course of atherosclerosis when it was only used in the last period of the experiment^12^. Consistent with these findings, our study demonstrated that rs1881457 in *IL13* was only associated with late-onset CAD, which might be due to the constantly decreasing IL-13 level, which in turn, might lead to the accumulation of glucose and a weakened anti-inflammatory response.

Men are more susceptible CAD than women, but no studies have explained the exact mechanism underlying this difference. In 1991, Bonnin *et al*. reported that male sex modifies the effect of smoking^34^. Subsequently, Liu *et al*. and Sadeghnejad *et al*. both discovered that the function of variants in *IL13* might be influenced by exposure to tobacco smoke^35,36^. Additionally, Chen *et al*. first reported that male gender can modify the interactions between variants in *IL13* and prenatal exposure to tobacco smoke in atopic diseases^37^. In our study, we found that rs1881457^C^ in *IL13* increased the risk of CAD in male patients. According to these findings, we hypothesized that, in males, rs1881457 in *IL13* might enhance the effect of prenatal exposure to tobacco smoke, which could then increase the risk of CAD. Thus, rs1881457 in *IL13* is a major cause of CAD in male subjects, and the underlying mechanism is the interaction of rs1881457 in *IL13* with prenatal exposure to tobacco smoke.

In summary, our study confirmed that two previous variants in *IL13* that were identified in a GWAS (rs2069744 and rs20541) were not associated with CAD in a large Chinese Han population. However, we discovered that a new variant in the regulatory region of *IL13*, i.e., rs1881457^C^, increased the CAD risk, and this result was lacking from the findings of the CARDIoGRAMplusC4D Consortium. We conclude that *IL13* might be involved in the development of CAD. Further studies with gene-gene and gene-environmental factor interaction analyses and larger sample sizes and functional studies in human tissues or cells are needed to provide more evidence about the exact functional mechanisms of IL-13 in CAD.

## Materials

### Populations

The subjects (1863 CAD cases and 1841 controls in total) were selected from the Union Hospital in Wuhan (Hubei, China). The criteria for enrollment as a CAD case were a diagnosis with percutaneous coronary intervention, myocardial infarction, coronary artery bypass graft, and/or ≥50% coronary stenosis in any one of the main vessels (i.e., the right coronary artery, left anterior descending, left main and left circumflex artery) on coronary angiography^38–40^. Individuals with coronary artery spasms, congenital heart disease, adolescent hypertension, valvular heart disease, type 1 diabetes, and serious renal or hepatic diseases were not included in this study.

According to previously published guidelines, we selected subjects with major coronary artery stenosis of no more than 30% and without histories of myocardial infarction, CAD, or ischemic stroke as controls^41^. Individuals who had coronary artery spasms, adolescent hypertension, type 1 diabetes, congenital heart disease, rheumatic autoimmune disease, tumor or stroke were not included. All of the participants’ clinical data, including age, BMI, gender, hypertension, smoking status and diabetes, were collected as detailed in previous studies^38–41^. The fasting TG, Tch, LDL-c and HDL-c concentrations were assessed with by standard methods^42,43^.

The DNA samples were collected using a purification kit (Wizard Genomic, Promega Corporation, Madison, WI)^38–41^. Coronary angiograms were available for a total of 1431 CAD individuals, and the coronary artery stenoses were assessed using the Gensini scores^44^. The coronary segment scores was judged as 32, 16, 8, 4, 2 or 1 for stenosis percentages of 100%, 99–91%, 90–76%, 75–51%, 50–26% or 25–0%, respectively. We then multiplied the score with a specific factor that was based on the vessel importance and size (ranging from 5.0 to 0.5). The Gensini index for each patient was the total sum of the weights of the segments.

This study followed the ethical principles of Declaration of Helsinki and passed a review by the ethics committee of Tongji Medical College, Huazhong University of Science and Technology. All participants signed an informed consent form of their own accord.

### Tag SNPs

We selected the tag SNPs as follows according to the following criteria: (1) the *r*^2^ value threshold of the LD between the SNPs was more than 0.8 (HapMap CHB and JPT data sets, v.3, release 2); (2) the threshold for the minor allele frequency (MAF) of the variant was greater than 0.05; and (3) the variant was functional or potentially functional (Promoter and Genevar). Three satisfactory variants (rs1881457, rs2069744 and rs20541) in *IL13* were selected for this association study (Fig. 1b). Rs1881457 is located in the promoter region of *IL13* and has been reported to regulate the expression of *IL13*^16,17^. The variants were genotyped using a high-resolution melt system (Rotor-gene 6000, Corbett Life Science, Concorde, NSW, Australia), with a 25-µl volume PCR reaction system as reported in our previous study^38–41^ using 0.5 μl of LC Green dye, 10 pmol of each primer (the forward primer was 5′-CGTGGGCCCTCTACTACAGA-3′, and the reverse primer was 5′-CCACACTCGAAGCTTCCCA-3′ for rs1881457; the forward primer was 5′-TGAGGTTAAGTGACAGAGGCT-3′ and the reverse primer was 5′-AAATGCCCTGCCTTCTGATG-3′ for rs2069744; and the forward primer was 5′-GGTGGCCCAGTTTGTAAAGG-3′ and the reverse primer was 5′-CAGGTCCTGTCTCTGCAAAT-3′ for rs20541), 30 ng of genomic DNA, and other contents^45^. The concordance rate of this genotyping method was 100%, and the results were confirmed by Sanger DNA sequencing.

### Statistics

Hardy-Weinberg disequilibrium tests in the control population were performed using PLINK software (version 1.07, Broad institute, USA). Pearson’s chi-square tests with 2 × 2 or 2 × 3 contingency tables were conducted in the allelic and genotypic association analyses (v.21.0, SPSS, Inc., Chicago, IL)^38,39^. Categorical data, including allele and haplotype frequencies, gender, smoking status, and other data, were tested using the chi-square test. Measurement data, such as BMI, age and blood lipid levels, were tested using *t*-tests. The traditional risk factors for CAD, including age, BMI, gender, hypertension, smoking, diabetes and lipid levels, were adjusted as covariates by logistic regression analysis (SPSS, v.21.0). The haplotype reconstruction and haplotype analysis were performed using Haploview (v.4.2, Informer Technologies, Inc.) or SPSS (v.21.0, Inc., Chicago, IL). Quartile case-control association analysis and liner regression analysis were utilized to test the associations between the levels of the Gensini scores and the SNPs. The correlations between the variant genotypes and the Gensini scores (CAD severity) was examined with Mann-Whitney *U* tests.

### Data availability

The datasets generated during and/or analyzed during the current study are available from the corresponding author on reasonable request.

## Electronic supplementary material

Supplementary Information
