# Supplementary material for: IL-13 may be involved in the development of CAD via different mechanisms under different conditions in a Chinese Han population
Source: Sci Rep. 2018 Apr 18;8:6182. doi: 10.1038/s41598-018-24592-9 (PMC5906444; doi:10.1038/s41598-018-24592-9)
Supplement: Supplementary file 1 — Supplementary Information [file 41598_2018_24592_MOESM1_ESM.pdf]

Supplementary Information

IL-13 may be involved in the development of CAD via different mechanisms under different conditions in a Chinese Han population

Ling-Feng Zha, Shao-Fang Nie, Qian-Wen Chen, Yu-Hua Liao, Hong-Song Zhang, Jiang-Tao Dong, Tian Xie, Fan Wang, Ting-Ting Tang, Ni Xia, Cheng-Qi Xu, Ying-Chao Zhou, Zhi-Peng Zeng, Jiao Jiao, Peng-Yun Wang, Qing K. Wang, Xin Tu, Xiang Cheng

Supplementary tables

Population	SNP-allele	Model	N		p_{obs}	p_{adj}	OR (95% CI)
			Case	Control			
Discovery-cohort	rs1881457[C]	ADD	62/243/383	49/259/422	0.262	0.495	1.09 (0.86-1.38)
		DOM	305/383	308/422	0.416	0.902	1.02 (0.75-1.39)
		REC	62/626	49/681	0.107	0.171	1.47 (0.85-2.56)
	rs2069744[T]	ADD	3/119/527	10/125/551	0.180	0.435	0.86 (0.59-1.25)
		DOM	122/527	135/551	0.683	0.764	0.94 (0.62-1.41)
		REC	3/646	10/676	0.064	0.044	0.18 (0.04-0.96)
	rs20541[A]	ADD	68/299/336	56/258/243	0.328	0.282	0.87 (0.67-1.13)
		DOM	367/336	314/243	0.140	0.604	0.91 (0.65-1.28)
		REC	68/635	56/501	0.822	0.131	0.64 (0.35-1.15)
Validation-cohort	rs1881457[C]	ADD	64/347/583	52/291/461	0.844	0.431	1.14 (0.82-1.59)
		DOM	411/583	343/461	0.575	0.644	1.10 (0.73-1.68)
		REC	64/930	52/752	0.980	0.299	1.52 (0.69-3.38)
	rs2069744[T]	ADD	8/109/700	6/153/730	0.072	0.579	1.17 (0.68-2.02)
		DOM	117/700	159/730	0.046	0.759	1.10 (0.61-1.96)
		REC	8/809	6/883	0.486	0.179	4.95 (0.48-50.8)
	rs20541[A]	ADD	71/353/369	90/468/412	0.226	0.643	0.92 (0.65-1.31)
		DOM	424/369	558/412	0.088	0.897	0.97 (0.63-1.50)
		REC	71/722	90/880	0.814	0.389	0.68 (0.28-1.65)
Combined-cohort	rs1881457[C]	ADD	126/590/966	101/550/883	0.585	0.386	1.08 (0.90-1.30)
		DOM	716/966	651/883	0.940	0.788	1.03 (0.82-1.30)
		REC	126/1556	101/1433	0.316	0.114	1.42 (0.92-2.18)

	ADD	11/228/1227	16/278/1281	0.209	0.381	0.88 (0.66-1.18)
rs2069744[T]	DOM	239/1227	294/1281	0.087	0.516	0.90 (0.66-1.23)
	REC	11/1455	16/1559	0.435	0.231	0.47 (0.14-1.62)
	ADD	139/652/705	146/726/655	0.059	0.344	0.91 (0.75-1.11)
rs20541[A]	DOM	791/705	872/655	0.019	0.626	0.94 (0.73-1.21)
	REC	139/1357	146/1381	0.800	0.205	0.75 (0.48-1.17)

Table S1. Genotypic association of the selected variants in *IL13* with CAD in the Chinese Han population. $p_{obs,}$ observed *p*-value; $p_{adj,}$ *p*-value adjusted for age, gender, BMI, hypertension, diabetes mellitus, smoking history, Tch, TG, HDL-c and LDL-c; OR, odds ratio after the adjustment; ADD, additive model, rs1881457_CC/AC/AA; rs2069744_TT/CT/CC; rs20541_AA/GA/GG; DOM, dominant model, rs1881457_CC+AC/AA; rs2069744_TT+CT/CC; rs20541_AA+GA/GG; REC, recessive model, rs1881457_CC/AC+AA; rs2069744_TT/CT+CC; rs20541_AA/GA+GG.

	Haplotype	N (%)		p_{obs}	p_{adj}	OR (95% CI)
		Case	Control			
***IL13* gene (rs1881457$^{C/A}$-rs2069744$^{T/C}$-rs20541$^{A/G}$)**	C-T-A	133 (5.5%)	161 (7.2%)	0.026	0.443	0.85 (0.55-1.30)
	C-T-G	24 (1.0%)	10 (0.4%)	0.036	0.772	1.18 (0.39-3.58)
	C-C-A	208 (8.6%)	201 (9.0%)	0.645	0.460	0.88 (0.62-1.24)
	C-C-G	218 (9.0%)	180 (8.0%)	0.314	0.484	1.14 (0.79-1.61)
	A-T-A	39 (1.6%)	33 (1.5%)	0.726	0.042	2.27 (1.03-5.00)
	A-T-G	20 (0.8%)	2 (0.1%)	0.003	0.019	9.09 (1.43-50.0)
	A-C-A	361 (14.9%)	347 (15.5%)	0.606	0.122	0.80 (0.60-1.06)
	A-C-G	1417 (58.6%)	1304 (58.3%)	-	-	-

Table S2. Haplotypic association analysis of *IL13* with CAD in the Chinese Han population. p_{obs}, observed *p*-value; p_{adj}, *p*-value adjusted for age, gender, BMI, hypertension, diabetes mellitus, smoking history, Tch, TG, HDL-c and LDL-c were adjusted; OR, odds ratio after the adjustment.

Population	SNP-allele	Model	Clinical-CAD		Anatomical-CAD	
			p_{adj}	OR (95% CI)	p_{adj}	OR (95% CI)
Combined-cohort	rs1881457^{C}	ALLE	0.280	1.11 (0.92-1.35)	0.261	1.12 (0.92-1.38)
		ADD	0.294	1.11 (0.92-1.34)	0.272	1.12 (0.92-1.37)
		DOM	0.588	1.07 (0.84-1.36)	0.437	1.11 (0.86-1.42)
		REC	0.131	1.42 (0.90-2.23)	0.238	1.34 (0.83-2.16)
	rs2069744^{T}	ALLE	0.596	0.92 (0.68-1.25)	0.425	0.88 (0.64-1.21)
		ADD	0.596	0.92 (0.68-1.25)	0.429	0.88 (0.64-1.21)
		DOM	0.754	0.95 (0.69-1.32)	0.498	0.89 (0.63-1.26)
		REC	0.300	0.50 (0.14-1.84)	0.492	0.64 (0.18-2.26)
	rs20541^{A}	ALLE	0.981	1.00 (0.82-1.22)	0.866	0.98 (0.80-1.21)
		ADD	0.981	1.00 (0.81-1.23)	0.860	0.98 (0.79-1.22)
		DOM	0.593	1.08 (0.82-1.41)	0.698	1.06 (0.80-1.40)
		REC	0.327	0.79 (0.50-1.26)	0.287	0.77 (0.47-1.25)

Table S3. Association analysis of the SNPs in *IL13* with CAD in the disease status subgroups. The anatomical-CAD group was defined as severe coronary stenosis and the clinical-CAD group is myocardial infarction or revascularization. p_{adj}, *p*-value adjusted for age, gender, BMI, hypertension, diabetes mellitus, smoking history, Tch, TG, HDL-c and LDL-c; OR, odds ratio after the adjustment.; ADD, additive model, rs1881457_CC/AC/AA; rs2069744_TT/CT/CC; rs20541_AA/GA/GG; DOM, dominant model, rs1881457_CC+AC/AA; rs2069744_TT+CT/CC; rs20541_AA+GA/GG; REC, recessive model, rs1881457_CC/AC+AA; rs2069744_TT/CT+CC; rs20541_AA/GA+GG.

SNP-allele	Model	Quantitative trait association				Case control association			
		beta	SE	r^2	p_{adj}	N		p_{adj}	OR (95%CI)
						1st	4th		
rs1881457[C]	ALLE	0.019	0.037	0.046	0.594	630	646	0.777	1.04 (0.80-1.35)
	ADD	0.019	0.036	0.047	0.603	315	323	0.786	1.04 (0.80-1.34)
	DOM	0.032	0.045	0.047	0.484	315	323	0.589	1.10 (0.79-1.52)
	REC	-0.007	0.088	0.046	0.934	315	323	0.735	0.90 (0.50-1.64)

Table S4. Association analysis of rs1881457 in *IL13* with the LN of Gensini scores. The 1st quartile was defined as the quartile with the lowest LN of Gensini scores, and the 4th quartile was defined as the quartile with the highest LN of Gensini scores. p_{obs}, observed p-value; p_{adj}, p-value adjusted for the covariates; OR, odds ratio after the adjustment; the p_{adj} values and OR values were obtained using a multivariate logistic regression analysis; ADD, additive model, rs1881457_CC/AC/AA; rs2069744_TT/CT/CC; rs20541_AA/GA/GG; DOM, dominant model, rs1881457_CC+AC/AA; rs2069744_TT+CT/CC; rs20541_AA+GA/GG; REC, recessive model, rs1881457_CC/AC+AA; rs2069744_TT/CT+CC; rs20541_AA/GA+GG.

Characteristics	Combined-cohort		
	**CAD early-onset (n=659)**	**CAD late-onset (n=1204)**	***p***
Age (years)	51.39±7.67	68.41±7.82	$<10^{-6}$
Male (%)	64.04	77.49	$<10^{-6}$
Smoking (%)	43.85	46.10	0.353
BMI (kg/m^2)	24.27±1.61	24.34±1.57	0.346
Hypertension (%)	59.03	72.59	$<10^{-6}$
DM (%)	28.53	34.39	0.010
Tch (mmol/L)	5.28±1.23	5.05±1.14	$8.1x10^{-5}$
TG (mmol/L)	2.12±1.52	1.65±1.04	$<10^{-6}$
HDL-c (mmol/L)	1.09±0.27	1.12±0.29	0.037
LDL-c (mmol/L)	3.16±1.13	2.95±1.01	$9.9x10^{-5}$
Gensini scores	30.57±23.99	33.04±24.76	0.064

Table S5. The characteristics of the subgroups. The data are shown as the mean ±SD. Categorical data, including allele frequency, gender, smoking status and other data, were tested using chi-square tests, and measurement data, such as BMI, age and blood lipid level, were tested using t-tests; CAD, coronary artery disease; DM, diabetes mellitus; BMI, body mass index; Tch, total cholesterol; TG, triglyceride; HDL-c, high-density lipoprotein cholesterol; LDL-c, low-density lipoprotein cholesterol. Age for the CAD is the age at diagnosis.

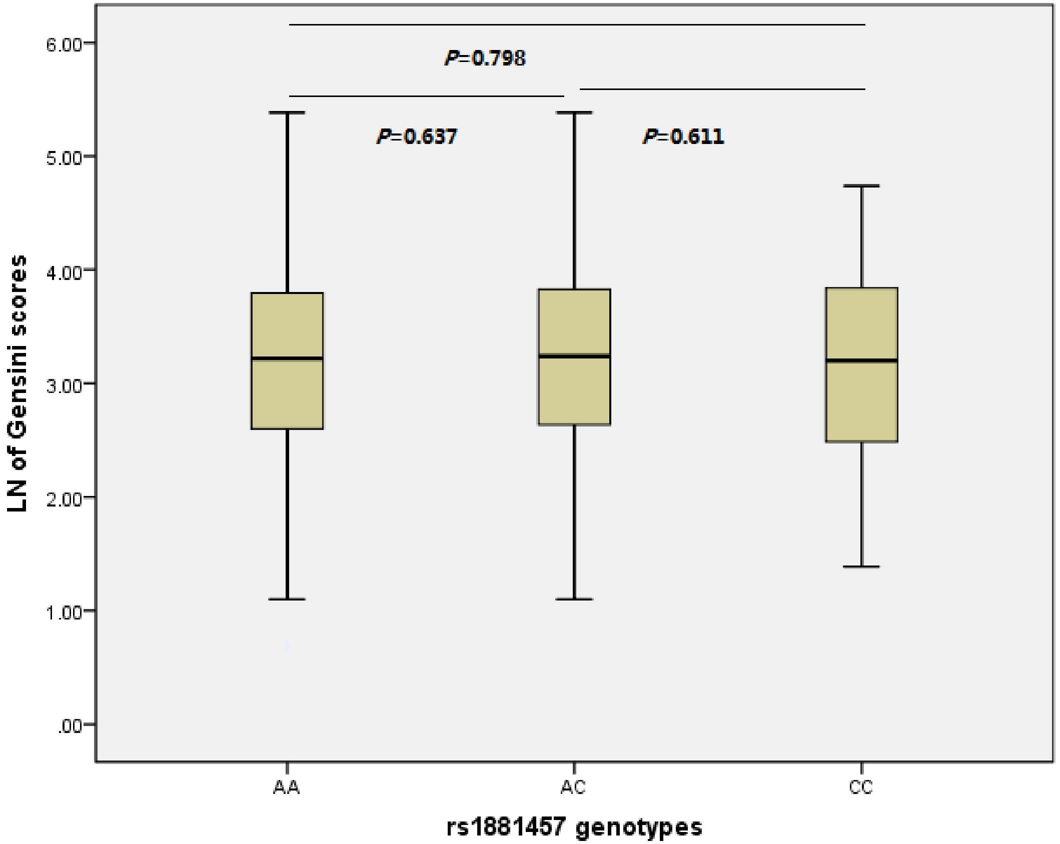

Figure S1. Association analysis between the LN of Gensini scores and the genotypes of rs1881457. The distribution difference of the LN of the Gensini scores in different genotypes of rs1881457 was compared by Mann-Whitney U-test.
